# ADHD Diagnosis and Timing of Medication Initiation Among Children Aged 3 to 5 Years

**DOI:** 10.1001/jamanetworkopen.2025.29610

**Published:** 2025-08-29

**Authors:** Yair Bannett, Ingrid Luo, Rodrigo Azuero-Dajud, Heidi M. Feldman, Farah W. Brink, Tanya E. Froehlich, Holly K. Harris, Kristin Kan, Kate E. Wallis, Kaitlin Whelan, Lisa Spector, Christopher B. Forrest

**Affiliations:** 1 Division of Developmental-Behavioral Pediatrics, Stanford University School of Medicine, Stanford, California; 2 Quantitative Sciences Unit, Stanford University School of Medicine, Stanford, California; 3 Department of Pediatrics, Children’s Hospital of Philadelphia, Perelman School of Medicine, University of Pennsylvania, Philadelphia; 4 Department of Pediatrics, Nationwide Children’s Hospital, The Ohio State University College of Medicine, Columbus; 5 Department of Pediatrics, Cincinnati Children’s Hospital Medical Center, University of Cincinnati College of Medicine, Cincinnati, Ohio; 6 Division of Developmental and Behavioral Pediatrics, Texas Children’s Hospital, Baylor College of Medicine, Houston; 7 Department of Pediatrics, Ann and Robert H. Lurie Children’s Hospital of Chicago, Chicago, Illinois; 8 Division of Developmental and Behavioral Pediatrics, Children’s Hospital of Philadelphia, Philadelphia, Pennsylvania; 9 Department of Pediatrics, University of Colorado, Aurora; 10 Division of Developmental and Behavioral Pediatrics, Nemours Children’s Hospital, Orlando, Florida

## Abstract

**Importance:**

Early identification and treatment of attention-deficit/hyperactivity disorder (ADHD) symptoms in preschool-age children is important for mitigating social, emotional, and academic problems. Clinical practice guidelines recommend first-line behavior intervention before considering medication treatment for children aged 4 to 5 years.

**Objective:**

To assess variation in rates of ADHD identification and rates and timing of medication initiation in children aged 3 to 5 years in primary care settings across 8 US pediatric health systems and to identify patient factors associated with the time from diagnosis to prescription.

**Design, Setting, and Participants:**

This retrospective cohort study used electronic health records from primary care clinics affiliated with 8 academic institutions participating in the PEDSnet Clinical Research Network. Participants were children aged 3 to 5 years seen between 2016 to 2023. Data were extracted from the PEDSnet database on April 18, 2025.

**Exposure:**

ADHD diagnosis at age 4 to 5 years.

**Main Outcomes and Measures:**

The primary outcomes were (1) rate of ADHD diagnosis, (2) rate of stimulant and nonstimulant prescription after diagnosis before age 7 years, and (3) time from first ADHD-related diagnosis (including symptom-level diagnoses) to medication prescription. Independent variables included institution, year of diagnosis, patient age, sex, race and ethnicity, medical insurance, and presence of comorbidities. Multivariable Cox proportional hazards models were used to estimate associations between clinical and demographic variables and time from diagnosis to prescription.

**Results:**

Of 712 478 children seen in primary care at age 3 to 5 years, 9708 (1.4%) received an ADHD diagnosis at age 4 to 5 years (range across institutions, 0.5%-3.1%; median [IQR] age at first ADHD-related diagnosis, 5.31 [4.86-5.66] years). Of those with ADHD, 7414 (76.4%) were male, 1762 (18.1%) were Hispanic, 122 (1.3%) were non-Hispanic Asian, 3014 (31.0%) were non-Hispanic Black, 479 (4.9%) were non-Hispanic multiracial, 3782 (39.0%) were non-Hispanic White, 148 (1.5%) were non-Hispanic other, and 401 (4.1%) were of unknown race and ethnicity. Of 9708 preschool-age children with ADHD, 6624 (68.2%) were prescribed ADHD medications before age 7 years, and 4092 (42.2%) were prescribed medications within 30 days of the first documentation of an ADHD-related diagnosis (range across institutions, 26.0%-49.0%). Asian (adjusted hazard ratio [aHR], 0.51; 95% CI, 0.38-0.68), Hispanic (aHR, 0.75; 95% CI, 0.70-0.81), and Black (aHR, 0.88; 95% CI, 0.83-0.94) children with ADHD were less likely to be prescribed medication early compared with White children. Older vs younger patients (aHR, 1.62; 95% CI, 1.55-1.69), male vs female patients (aHR, 1.17; 95% CI, 1.11-1.25), and publicly insured vs privately insured patients (aHR, 1.09; 95% CI, 1.03-1.15) were more likely to be prescribed medication early.

**Conclusion and Relevance:**

In this retrospective cohort study of preschool-age children with ADHD seen in primary care in 8 large pediatric health systems, many children were prescribed medications at or shortly after the first documented diagnosis. Analysis of clinical documentation is needed to understand early prescription patterns.

## Key Points

**Question:**

What is the prevalence of attention-deficit/hyperactivity disorder (ADHD) diagnoses and timing of initiation of medications among children aged 3 to 5 years seen in primary care?

**Findings:**

In this cohort study of electronic health records for 712 478 children seen in primary care practices at 8 US pediatric health systems, the prevalence of ADHD was 1.4% (range across institutions, 0.5%-3.1%); 68.2% of patients were prescribed medications and 42.2% had medications prescribed within 30 days of diagnosis, with variation by race, ethnicity, and insurance type.

**Meaning:**

These findings suggest that investigation of barriers to nonpharmacological interventions, as well as factors associated with early prescriptions of ADHD medications, for preschool-age children is warranted.

## Teaser

This cohort study examines variations in rates of attention-deficit/hyperactivity disorder (ADHD) diagnosis and rates and timing of medication initiation among children aged 3 to 5 years.

## Introduction

Attention-deficit/hyperactivity disorder (ADHD) is a highly prevalent neurodevelopmental disorder, estimated to affect 10% of US children.^1^ ADHD is increasingly being diagnosed in children before they enter school. A national survey in 2022 found that 2.4% of children aged 3 to 5 years had a diagnosis of ADHD.^2^ Preschool-age children with symptoms of ADHD are at risk for social and emotional problems and academic failure.^3,4^ Recognizing the early onset of ADHD, the American Academy of Pediatrics subcommittee published updated evidence-based clinical practice guidelines for primary care management of ADHD in 2011^5^ and reaffirmed in 2019.^6^ These guidelines include a separate set of recommendations for management of ADHD and ADHD symptoms in preschool-age children (aged 4-5 years). The guidelines recommend that primary care pediatricians (PCPs) start with parent training in behavior management and then consider medication treatment with methylphenidate as a second-line treatment, given stronger evidence for behavioral intervention than for medications in this age group.^7^

Most US children with ADHD first receive a diagnosis from and are treated by their PCP.^8,9,10^ However, to date, very few studies have assessed primary care management of ADHD in this young age group.^11^ Survey-based studies^2,12,13^ have found variation across patient subgroups in parent-reported ADHD diagnosis and treatment rates in preschool-age children. Prior claims-based and electronic health record (EHR)–based studies^14,15,16,17^ have assessed trends in prescription rates of stimulants in preschool-age children but did not assess the timing of treatment in relation to the initial diagnosis. Furthermore, information on variation in ADHD diagnosis and treatment across US health systems is limited, and evidence of sociodemographic disparities in diagnosis and treatment is mixed.^10,18,19,20,21,22^ To our knowledge, there are no large multisite studies that leveraged clinical data from EHRs to assess rates of PCP diagnosis and treatment of ADHD in preschool-age children, including adherence to practice guidelines^5,6^ in the timing of medication treatment, and differences in clinical care across sociodemographic groups.

In this study, we assessed rates of ADHD identification and medication prescription and timing of medication treatment in children aged 3 to 5 years in primary care settings across 8 large US pediatric health systems. We also evaluated patient factors associated with the time from first diagnosis to prescription.

## Methods

We present this cohort study in accordance with the Strengthening the Reporting of Observational Studies in Epidemiology (STROBE) reporting guidelines.^23^ This study was approved by a multisite single institutional review board led by Children’s Hospital of Philadelphia, which waived the need for informed consent because all data were deidentified, in accordance with 45 CFR §46.

### Setting and Data Source

Eight institutions that participate in PEDSnet, a national pediatric learning health system, provided EHR data for this study. Participating institutions included Children’s Hospital of Colorado (Aurora, Colorado), Children’s Hospital of Philadelphia (Philadelphia, Pennsylvania), Cincinnati Children’s Hospital (Cincinnati, Ohio), Lurie Children’s Hospital of Chicago (Chicago, Illinois), Nationwide Children’s Hospital (Columbus, Ohio), Nemours Children’s Health (Wilmington, Delaware, and Orlando, Florida), Packard Children’s Hospital Stanford (Stanford, California), and Texas Children’s Hospital (Houston, Texas). Institutions extract data on a quarterly basis from source EHR systems and transform the data to the Observational Medical Outcomes Partnership data model (currently version 5.7). Data are uploaded to the PEDSnet Coordinating Center at the Children’s Hospital of Philadelphia, where they undergo rigorous data quality assessment. For this study, we extracted data from version v56 of the PEDSnet database on April 18, 2025.

### Study Design and Cohort Selection

This was a retrospective cohort study of EHR data from all encounters (office, telehealth, telephone, and administrative) of children aged 3 to 5 years seen between January 1, 2016, to December 31, 2023, in primary care clinics affiliated with 8 academic institutions. We included children who were seen 2 or more times with at least 6 months of follow-up. Primary care clinics were identified using a procedure validated in 1 institution, requiring (1) 5% or more well-care visits among all visits, (2) 1 or more childhood immunization procedure code, and (3) removing care sites whose names were null or included the word *laboratory* (eTable 1 in Supplement 1).

We defined an ADHD-related encounter as an encounter with ADHD diagnoses or medication prescriptions (stimulants included methylphenidate or amphetamines; nonstimulants included α-agonists or atomoxetine). In a prior study,^11^ we found that PCPs frequently used symptom-level diagnoses of ADHD (eg, hyperactivity) in children younger than 6 years. Accordingly, we defined 2 types of ADHD diagnoses: (1) disorder level (eg, ADHD combined subtype) and (2) symptom level (eg, hyperactivity or inattention). Patients were included in the ADHD cohort if they received at least 1 disorder-level ADHD diagnosis between ages 4 and 5 years because guidelines recommend considering an ADHD diagnosis starting from age 4 years. ADHD medications prescribed after the initial diagnosis and before age 7 years were included.

To capture PCP documentation of common developmental and/or behavioral comorbid conditions, as recommended by practice guidelines, we extracted for each patient any visit diagnosis of comorbid conditions (autism, anxiety, depression, disruptive behavior disorder, global developmental delay or intellectual disability, language delay or disorder, learning problem or disability, and sleep problems) documented at or after the initial ADHD diagnosis. eTables 2 and 3 in Supplement 1 detail code sets for ADHD diagnoses, ADHD medications, and comorbid diagnoses.

### Study Outcomes and Measures

Primary outcomes included (1) rate of ADHD diagnosis (disorder level), (2) rate of stimulant and nonstimulant prescription after the initial diagnosis (disorder or symptom level) and before age 7 years, and (3) time from initial ADHD diagnosis (disorder or symptom level) to prescription (days). Secondary outcomes included (1) follow-up of prescribed patients within 60 days (ADHD-related encounter) and (2) documentation of comorbid conditions.

Patient sociodemographic and clinical characteristics documented in the EHR included age (continuous, at encounter of interest), sex, race and ethnicity (Asian non-Hispanic, Black non-Hispanic, Hispanic irrespective of race, White non-Hispanic, multiracial non-Hispanic, other non-Hispanic [ie, American Indian or Alaska Native, Native Hawaiian or other Pacific Islander, and patients marked as other in the EHR source data], and unknown), medical insurance at first ADHD-related encounter (private, public, or other), presence of specific developmental or behavioral comorbid condition, and number of primary care visits during the study period. Data on race and ethnicity are included in this study because prior studies^10,18,19,20,21,22^ suggested that they may contribute to disparities in diagnosis and treatment of children with ADHD.

### Statistical Analysis

Patient characteristics were summarized using frequencies (percentages) for categorical variables and median (IQR) for continuous variables. Values less than 11 were censored for confidentiality.

Time to first prescription was measured from the patient’s first ADHD diagnosis (disorder or symptom level) after age 3 years to before age 7 years. For descriptive analysis, time to prescription was categorized as at or shortly after diagnosis (0-30 days), 1 to 6 months from diagnosis, and more than 6 months after diagnosis, a minimum time frame recommended in the 2011 practice guidelines^5^ to pursue first-line behavioral treatment. We used inverse probability treatment weighting with cumulative incidence functions to visualize racial and ethnic differences while accounting for potential confounding by primary care utilization and care institution. Assumptions of positivity and between-patient independence were evaluated and met. We then fit multivariable Cox proportional hazards models to estimate associations between clinical and demographic variables (ie, initial diagnosis type [disorder level vs symptom level], specific comorbidities, EHR-recorded race and ethnicity, age at diagnosis, sex, and insurance type) and time to prescription, adjusting for year of diagnosis, primary care utilization (categorized as low [<5 visits], medium [5–10 visits], and high [>10 visits]), and institution. For comorbidities, we selected autism, global developmental delay or intellectual disability, sleep problems, and disruptive behavioral disorder because we hypothesized these conditions may influence the decision to prescribe medications. The proportional hazards assumption was tested using Schoenfeld residuals and was met. Patients were censored at their last follow-up encounter if they did not receive a prescription before age 7 years.

Time from prescription to follow-up was visualized using cumulative incidence curves, presented overall and for each type of follow-up (in-person or telehealth) separately. Institutional variation in comorbidity documentation was shown using box plots for any comorbidity documentation and for individual conditions.

In sensitivity analyses, first we changed the definition of first ADHD-related encounter to include nonspecific diagnosis codes of behavior problems (eTable 4 in Supplement 1), to assess the extent to which time to prescription is prolonged under the assumption that PCPs may use nonspecific diagnoses (eg, behavior concern) as the first indication of ADHD-related concerns. Using paired *t* tests, we compared time to prescription within the same patients under both definitions. Second, we stratified time to prescription by initial ADHD diagnosis type because the index event for measuring time to prescription was a disorder-level diagnosis in some patients and symptom-level diagnosis in others. Using cumulative incidence curves with log-rank tests, we tested the hypothesis that when PCPs document symptom-level diagnoses first, time to prescription is longer than when they document disorder-level diagnoses first. Third, we repeated all analyses excluding 1 institution with high rates of missing insurance data. All analyses were conducted using R statistical software version 4.3.2 (R Project for Statistical Computing). Statistical significance was defined as 2-sided *P* < .05.

## Results

**Figure 1.**
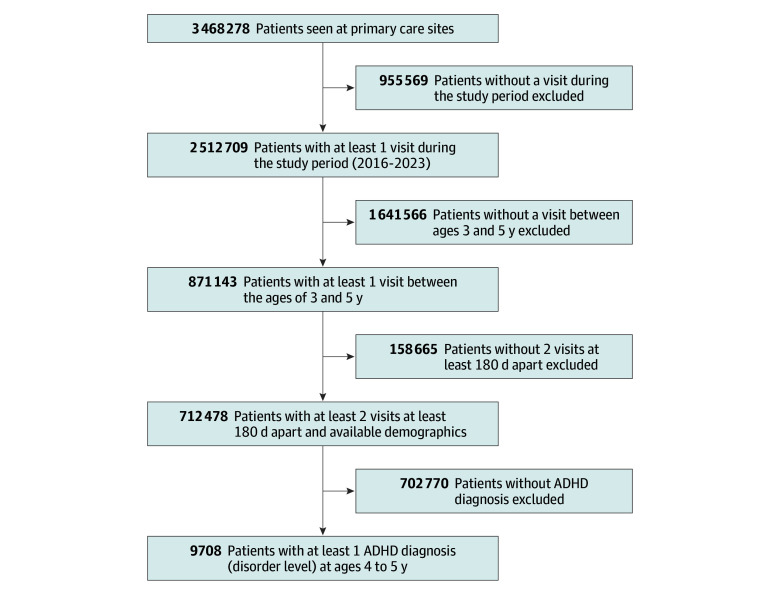
Participant Enrollment Flowchart ADHD indicates attention-deficit/hyperactivity disorder.

Of 712 478 children seen in primary care at ages 3 to 5 years, 9708 (1.4%) had an ADHD diagnosis (disorder level) at ages 4 to 5 years (Figure 1). The rate of ADHD diagnosis was variable across institutions (0.5%-3.1%) (eTable 5 in Supplement 1). The median (IQR) age at first ADHD-related diagnosis was 5.31 (4.86-5.66) years. Of 9708 children with ADHD, 7414 (76.4%) were male, 1762 (18.1%) were Hispanic, 122 (1.3%) were non-Hispanic Asian, 3014 (31.0%) were non-Hispanic Black, 479 (4.9%) were non-Hispanic multiracial, 3782 (39.0%) were non-Hispanic White, 148 (1.5%) were non-Hispanic other, and 401 (4.1%) were of unknown race and ethnicity; 6624 (68.2%) were prescribed medications for ADHD, including stimulants (5131 children [77.5% of prescribed children]), nonstimulants (1108 children [16.7%]), and both (385 children [5.8%]). Rates of ADHD medication prescriptions varied across institutions (44.1%-74.1% of children) (Table).

**Table.** Demographic and Clinical Characteristics of Children With ADHD, by Site

Characteristic	Patients, No. (%)^a^								
	Site A (n = 3857)	Site B (n = 1886)	Site C (n = 1672)	Site D (n = 934)	Site E (n = 918)	Site F (n = 202)	Site G (n = 127)	Site H (n = 112)	Overall (N = 9708)
Age, median (IQR), y									
At first encounter^b^	3.32 (3.03-4.50)	3.50 (3.06-4.72)	3.22 (3.04-4.39)	3.62 (3.15-4.70)	3.62 (3.13-4.68)	3.70 (3.06-4.67)	3.27 (3.06-4.27)	3.36 (3.07-4.45)	3.39 (3.05-4.57)
At initial ADHD-related diagnosis	5.28 (4.83-5.64)	5.29 (4.77-5.64)	5.27 (4.84-5.67)	5.47 (5.09-5.75)	5.38 (5.01-5.69)	5.28 (4.94-5.62)	5.19 (4.55-5.53)	5.38 (4.93-5.71)	5.31 (4.86-5.66)
Sex									
Female	871 (22.6)	467 (24.8)	378 (22.6)	252 (27.0)	237 (25.8)	41 (20.3)	29 (22.8)	19 (17.0)	2294 (23.6)
Male	2986 (77.4)	1419 (75.2)	1294 (77.4)	682 (73.0)	681 (74.2)	161 (79.7)	98 (77.2)	93 (83.0)	7414 (76.4)
Race and ethnicity									
Hispanic	966 (25.0)	394 (20.9)	167 (10.0)	76 (8.1)	36 (3.9)	22 (10.9)	53 (41.7)	48 (42.9)	1762 (18.1)
Non-Hispanic Asian	55 (1.4)	11 (0.6)	<11	11 (1.2)	0	24 (11.9)	6 (4.7)	6 (5.4)	122 (1.3)
Non-Hispanic Black	1075 (27.9)	460 (24.4)	535 (32.0)	402 (43.0)	460 (50.1)	<11	46 (36.2)	26 (23.2)	3014 (31.0)
Non-Hispanic multiracial	120 (3.1)	75 (4.0)	96 (5.7)	126 (13.5)	44 (4.8)	<11	<11	<11	479 (4.9)
Non-Hispanic White	1418 (36.8)	841 (44.6)	743 (44.4)	307 (32.9)	370 (40.3)	68 (33.7)	15 (11.8)	20 (17.9)	3782 (39.0)
Non-Hispanic other^c^	56 (1.5)	65 (3.4)	<11	<11	<11	16 (7.9)	<11	<11	148 (1.5)
Unknown	167 (4.3)	40 (2.1)	121 (7.2)	12 (1.3)	<11	53 (26.2)	<11	<11	401 (4.1)
Insurance plan									
Private	1797 (46.6)	679 (36.0)	743 (44.4)	114 (12.2)	<11	149 (73.8)	13 (10.2)	18 (16.1)	3520 (36.3)
Public	1912 (49.6)	1202 (63.7)	901 (53.9)	802 (85.9)	80 (8.7)	53 (26.2)	98 (77.2)	91 (81.3)	5139 (52.9)
Other or unknown	148 (3.8)	<11	28 (1.7)	18 (1.9)	831 (90.5)	<11	16 (12.6)	<11	1049 (10.8)
Prescribed medication before age 7 y									
Yes	2857 (74.1)	1165 (61.8)	1170 (70.0)	588 (63.0)	625 (68.1)	103 (51.0)	56 (44.1)	60 (53.6)	6624 (68.2)
≤30 d	1805 (46.8)	625 (33.1)	666 (39.8)	409 (43.8)	450 (49.0)	57 (28.2)	33 (26.0)	47 (42.0)	4092 (42.2)
30-183 d	474 (12.3)	255 (13.5)	192 (11.5)	112 (12.0)	91 (9.9)	20 (9.9)	<11	<11	1159 (11.9)
>183 d (6 mo)	578 (15.0)	285 (15.1)	312 (18.7)	67 (7.2)	84 (9.2)	26 (12.9)	14 (11.0)	<11	1373 (14.1)
No	1000 (25.9)	721 (38.2)	502 (30.0)	346 (37.0)	293 (31.9)	99 (49.0)	71 (55.9)	52 (46.4)	3084 (31.8)
Age at first prescription, median (IQR), y	5.50 (5.09-5.82)	5.56 (5.16-5.89)	5.56 (5.14-5.89)	5.65 (5.36-5.88)	5.56 (5.21-5.84)	5.59 (5.21-5.89)	5.50 (5.10-5.78)	5.55 (5.33-5.90)	5.55 (5.15-5.85)
First prescription type^d^									
Stimulant	2278 (79.7)	872 (74.8)	826 (70.6)	507 (86.2)	474 (75.8)	90 (87.4)	32 (57.1)	52 (86.7)	5131 (77.5)
Nonstimulant	438 (15.3)	244 (20.9)	213 (18.2)	56 (9.5)	116 (18.6)	11 (10.7)	24 (42.9)	<11	1108 (16.7)
Both	141 (4.9)	49 (4.2)	131 (11.2)	25 (4.3)	35 (5.6)	<11	<11	<11	385 (5.8)

Abbreviation: ADHD, attention-deficit/hyperactivity disorder.

^a^ Cells with patient count less than 11 were censored, and percentages were not calculated.

^b^ Age at first encounter is the child’s age at the first primary care encounter after the child turned 3 years old.

^c^ Non-Hispanic other includes American Indian or Alaska Native, Native Hawaiian or other Pacific Islander, and patients marked as other in the electronic health record source data.

^d^ The denominator is patients who received ADHD medication prescriptions.

### Timing of Medication Treatment

**Figure 2.**
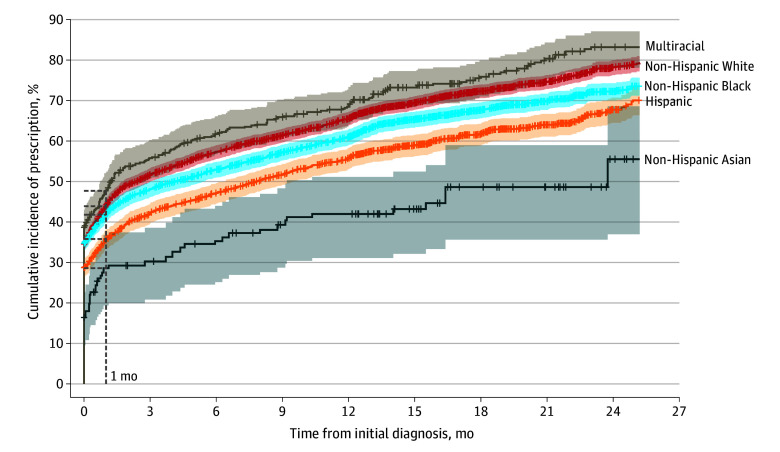
Time From Initial Attention-Deficit/Hyperactivity Disorder (ADHD) Diagnosis to Prescription by Race and Ethnicity Graph shows cumulative incidence of ADHD medication prescription by race and ethnicity with 95% CIs (shaded areas). The maximum time to prescription was 47 months; x-axis is limited to 24 months for visual clarity. Vertical ticks represent right-censoring events for patients who did not receive prescriptions before age 7 years, censored at their last follow-up date. At 1 month after diagnosis, incidence was highest for non-Hispanic multiracial children (47.7%) and lowest for non-Hispanic Asian children (28.6%). Analysis excluded patients with other or unknown race or ethnicity.

Of 9708 children aged 3 to 5 years with ADHD, 4092 (42.2%) were prescribed medications within a month of the initial ADHD-related diagnosis (disorder or symptom level; range across institutions, 26.0%-49.0%) compared with 1373 (14.1%) prescribed more than 6 months after the initial diagnosis (Table). The median (IQR) time from diagnosis to prescription varied by the child’s age at time of initial ADHD-related diagnosis: 390.5 (204.2-672) days for 264 children aged 3 years, 28.0 (0.0-289.2) days for 1550 children aged 4 years, and 0.0 (0.0-55.0) days for 4810 children aged 5 years (eTable 6 in Supplement 1). The time from diagnosis to prescription varied widely across racial and ethnic subgroups (Figure 2). At or shortly after diagnosis (0-30 days), the cumulative incidence of medication prescription among patients with ADHD was highest for multiracial children (47.7%; 95% CI, 42.8%-52.2%), non-Hispanic White children (43.9%; 95% CI, 42.3%-45.6%), and non-Hispanic Black children (41.8%; 95% CI, 39.9%-43.6%), and lower for Hispanic children (35.8%; 95% CI, 33.4%-38.1%) and non-Hispanic Asian children (28.6%; 95% CI, 19.4%-36.7%). At 2 years after diagnosis (450 person-years), prescription rates were high for non-Hispanic White children (78.2%; 95% CI, 76.3%-80.0%) and multiracial children (83.2%; 95% CI, 78.1%-87.1%), lower for non-Hispanic Black children (72.2%; 95% CI, 70.0%-74.3%) and Hispanic children (67.8%; 95% CI, 64.5%-70.7%), and substantially lower for non-Hispanic Asian children (55.6%; 95% CI, 37.0%-68.6%).

### Regression Model

**Figure 3.**
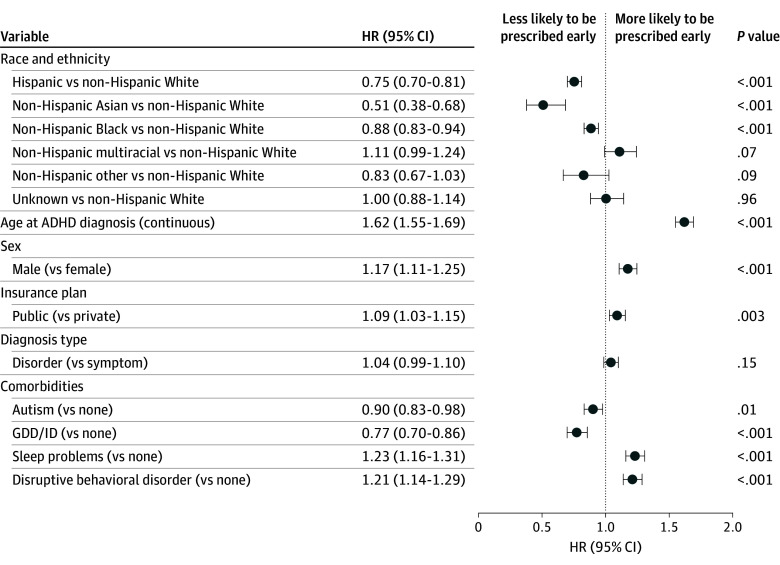
Adjusted Cox Regression Model Results Model was adjusted for year of diagnosis, initial diagnosis type (disorder vs symptom level), primary care utilization (low, <5 visits; medium, 5-10 visits; and high, >10 visits), presence of comorbidities, and institution. ADHD indicates attention-deficit/hyperactivity disorder; GDD/ID, global developmental delay/intellectual disability; HR, hazard ratio.

Cox regression model results examining time to first medication prescription after initial diagnosis and before age 7 years are presented in Figure 3. After adjusting for year of diagnosis, primary care utilization, and institution, Asian (adjusted hazard ratio [aHR], 0.51; 95% CI, 0.38-0.68), Hispanic (aHR, 0.75; 95% CI, 0.70-0.81), and Black (aHR, 0.88; 95% CI, 0.83-0.94) patients with ADHD were less likely to be prescribed medication early compared with non-Hispanic White patients. Older vs younger patients (aHR, 1.62; 95% CI, 1.55-1.69), male vs female patients (aHR, 1.17; 95% CI, 1.11-1.25), and publicly insured vs privately insured patients (aHR, 1.09; 95% CI, 1.03-1.15) were more likely to be prescribed medication early. Patients with sleep problems (aHR, 1.23; 95% CI, 1.16-1.31) and patients with disruptive behavior disorders (aHR 1.21; 95% CI, 1.14-1.29) were more likely to be prescribed early compared with those without these diagnoses.

### Secondary Outcomes

Of 6624 preschool-age children prescribed ADHD medications, 2684 (40.5%) had an ADHD-related encounter within 2 months of the first prescription (range across institutions, 25.9%-56.0%). Follow-up visits were mostly in person and less commonly via telehealth (eFigure 1 in Supplement 1). Data on rate of patient follow-up via telephone or secure messaging were not available.

**Figure 4.**
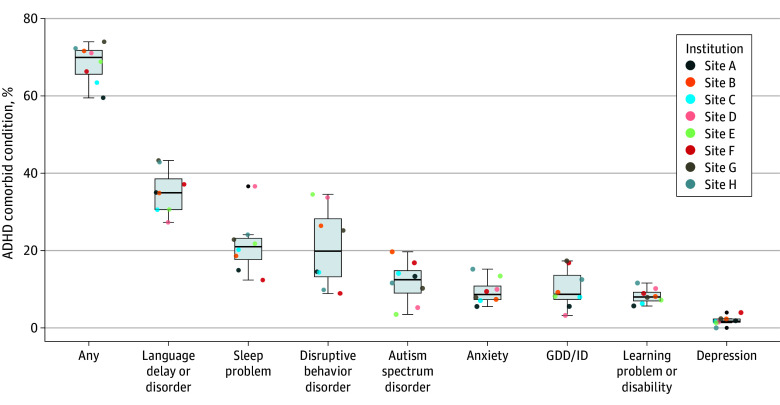
Documented Developmental or Behavioral Comorbid Conditions Among Children Aged 3 to 5 Years With Attention-Deficit/Hyperactivity Disorder (ADHD) Each box displays the 25th percentile (bottoms of boxes) to 75th percentile (tops of boxes) with medians (center lines); whiskers extend to data within 1.5 times the IQR, and dots represent institutional data points. GDD/ID indicates global developmental delay/intellectual disability.

PCPs documented comorbid conditions, as recommended by practice guidelines,^5,6^ in 65.0% of patients (6313 children) with low variability (coefficient of variation, 7.44%) across institutions (Figure 4). The most common comorbid conditions documented were language delay or disorder (3234 children [33.3%]), sleep problems (1887 children [19.4%]), and disruptive behavior disorders (1864 children [19.2%]).

### Sensitivity Analysis

When including nonspecific diagnosis codes of behavior problems as the first ADHD-related concern, 27.8% of patients (2696 children) were prescribed within 1 month of first concern, whereas 25.9% (2519 children) were prescribed after more than 6 months. Time to prescription was significantly longer when measured from first concern vs first ADHD-related diagnosis (median [IQR] difference, 0.0 [0.0-4.0] months; paired Wilcoxon signed-rank test, *P* < .001). Among 7730 children whose first ADHD-related diagnosis was disorder level, the estimated 30-day cumulative incidence of prescription was 47.7% (95% CI, 46.6%-48.8%), compared with 22.9% (95% CI, 21.1%-24.8%) for those whose initial diagnosis was symptom level. The cumulative incidence patterns differed significantly between diagnosis types (log-rank test, χ^2^ = 5.50; *P* = .02), with initially higher prescription rates for disorder-level diagnoses that converged after 1 year (eFigure 2 in Supplement 1). Excluding 1 institution that had high rates of missing insurance data did not change the results substantively (data not shown).

## Discussion

This cohort study revealed high variability across US pediatric health systems in rates of identification and medication treatment of children aged 3 to 5 years with ADHD. Overall, approximately two-thirds (68.2%) of preschool-age children with ADHD seen in primary care were prescribed ADHD medications before age 7 years, with 42.2% of children prescribed within 30 days of their initial ADHD-related diagnosis, contrary to practice guidelines^5,6^ that recommend first starting with evidence-based nonpharmacological intervention. We found evidence for disparities in care with high rates of early medication treatment among White children and those with public insurance. These findings highlight the need to investigate factors influencing early medication treatment of preschoolers with ADHD, especially in specific patient subgroups.

The prevalence of ADHD diagnosis varied across the 8 pediatric health systems in this study, aligning with Centers for Disease Control and Prevention survey-based data showing high variation in ADHD prevalence across US states.^24^ We found low rates of ADHD diagnosis in Hispanic, Asian, and Black children compared with White children, and high rates of diagnosis in publicly insured patients compared with privately insured patients, following previously reported trends.^12,13,20,25^

The high variation we found across institutions in the rate and timing of ADHD medication prescriptions highlights differences in clinical practice in the treatment of preschoolers with ADHD. Overall, medication prescription rates (44.1%-74.1% of patients across institutions) were higher than we had expected. Although no clear benchmark exists for the rate of preschool-age children with ADHD who should receive medications, clinical practice guidelines recommend medications as second-line treatment in cases with substantial dysfunction or lack of response to behavioral treatment. The rate of preschool-age children with ADHD prescribed medication by PCPs in the current study (68.2%) was higher than the rate prescribed by subspecialists in a prior study (46%),^26^ even though subspecialists often manage children with complex or severe presentations. Previous studies^17,20,27,28^ also reported that receipt of medication treatment was more common than first-line behavior treatment in US preschoolers. Future studies should investigate whether differences in clinical presentation, PCP knowledge of and patient access to nonpharmacological interventions, or family and PCP preferences are associated with high rates of medication treatment.

Among children who received a diagnosis of ADHD at ages 3 to 5 years, 42.2% were prescribed an ADHD medication within 30 days of the initial ADHD-related diagnosis (including symptom-level descriptors). Hence, more than one-third of patients lacked sufficient time for an evidence-based behavioral treatment before starting medications. This finding that, to our knowledge, has not been previously reported highlights the need to investigate factors that influence early medication prescriptions. In sensitivity analyses, rates of early prescription were lower both when using nonspecific behavior problems as the initial diagnosis (27.8%) and when including only symptom-level diagnoses (22.9%), suggesting that expanding the scope of investigation beyond diagnosis codes is necessary. Analyzing clinical free-text documentation may enhance our ability to outline treatment timelines of young children with ADHD, including timing of first concern, severity of symptoms, and recommendations for nonpharmacological interventions.

The high rates of early prescription that we found among both White children and those with public insurance suggests that multiple factors are associated with the timing of medication prescriptions. Prior literature^29,30^ found that minoritized racial and ethnic groups are less likely to initiate ADHD medication treatment compared with White patients because of negative attitudes about ADHD medications; however, these same groups are also less likely to initiate and engage in parent behavioral training. Publicly insured patients often encounter systemic barriers to accessing subspecialists and evidence-based parent behavioral training,^30,31^ which may motivate the clinician and the family to try medications early. Future interventions must address family-level, clinician-level, and system-level barriers to nonpharmacological treatments that may contribute, especially in certain subpopulations, to high rates of pharmacological treatment and low rates of nonpharmacological treatment of young children with ADHD.

We have also contributed to the limited literature on current rates of prescribing nonstimulants to treat preschool ADHD in primary care. Although there is limited evidence of the efficacy of nonstimulants in children younger than 6 years, prior studies^26,32^ found that subspecialists prescribed nonstimulants to approximately one-third of preschool-age children with ADHD, somewhat higher than PCP prescription rate in the present study.

The proportion of preschoolers with ADHD who were seen for follow-up within 2 months of medication prescription varied across institutions (25.9%-56.0%). A prior single-site study^11^ found a similarly low rate of follow-up in preschoolers prescribed ADHD medications. However, these rates are likely an underestimate of actual follow-up rates. As previously reported,^33^ children with ADHD are frequently followed in primary care by telephone or secure messaging—methods that have been shown to contribute to achieving symptom improvement without an in-person visit. In addition, children may be referred to subspecialists who assume responsibility for follow-up care. Rates of documentation of comorbid conditions were high (approximately two-thirds of patients), suggesting that PCPs adhere to guidelines in detecting common comorbid conditions in young children with ADHD.

### Limitations

Several limitations should be acknowledged. Study cohort identification was based on diagnostic codes, which carries the potential for misclassification error. Although we included symptom-level diagnoses of ADHD as the initial diagnosis, in some cases, that first diagnosis may not represent the first time that ADHD-related concerns were raised. For some patients, nonstimulant medications may have been prescribed to address sleep problems, rather than ADHD symptoms. Furthermore, information on the rate of PCP recommendation for first-line behavioral treatment, typically documented as free text, was not available to us.^28,34^ Therefore, we could not determine whether in some cases behavioral treatment was not available and, as recommended in guidelines, the PCP and family chose early medication treatment after weighing the risks and benefits. Future studies will need to extract information from clinical notes, which can be facilitated by natural language processing,^35,36^ to capture PCP recommendations about behavioral treatments, clinical reasoning behind early prescriptions, and the involvement of subspecialists in medication decisions.

## Conclusions

This large multisite study found high variation in the identification and treatment of ADHD in children aged 3 to 5 years who presented in primary care settings. The high rate of medication prescriptions among preschool-age children with ADHD and the lack of delay between initial diagnosis and prescription require further investigation to assess the appropriateness of early medication treatment. The variation in care among minoritized racial and ethnic groups uncovers disparities in care that require further exploration. Future studies will assess factors influencing prescribing patterns, including PCP and family preferences, severity of symptoms, and access to first-line nonpharmacological treatment across patient subgroups.

## Notes

Supplement 1. **eTable 1.** List of codes used for well care visits and immunizations **eTable 2.** List of codes used for ADHD diagnosis and ADHD medications **eTable 3.** List of codes used for developmental/behavioral comorbid conditions **eTable 4.** List of codes for behavioral problems used in sensitivity analysis **eTable 5.** Patient demographics of children seen in primary care at ages 3-5 years across 8 institutions (n=712,478) **eTable 6.** Time from diagnosis to prescription among preschool-age children with ADHD, stratified by patient age at time of initial ADHD-related diagnosis **eFigure 1.** Follow up of children 3-5 years who were prescribed ADHD medications **eFigure 2.** Time from initial ADHD diagnosis to medication prescription, stratified by the type of initial ADHD diagnosis (symptom-level first vs. disorder-level first)

Supplement 2.
Data Sharing Statement
