# Supplementary material for: ADHD Diagnosis and Timing of Medication Initiation Among Children Aged 3 to 5 Years
Source: JAMA Netw Open. 2025 Aug 29;8(8):e2529610. doi: 10.1001/jamanetworkopen.2025.29610 (PMC12397892; doi:10.1001/jamanetworkopen.2025.29610)
Supplement: Supplement 1. — eTable 1. List of codes used for well care visits and immunizations eTable 2. List of codes used for ADHD diagnosis and ADHD medications eTable 3. List of codes used for developmental/behavioral comorbid conditions eTable 4. List of codes for behavioral problems used in sensitivity analysis eTable 5. Patient demographics of children seen in primary care at ages 3-5 years across 8 institutions (n=712,478) eTable 6. Time from diagnosis to prescription among preschool-age children with ADHD, stratified by patient age at time of initial ADHD-related diagnosis eFigure 1. Follow up of children 3-5 years who were prescribed ADHD medications eFigure 2. Time from initial ADHD diagnosis to medication prescription, stratified by the type of initial ADHD diagnosis (symptom-level first vs. disorder-level first) [file jamanetwopen-e2529610-s001.pdf]

Supplemental Online Content

Bannett Y, Luo I, Azuero-dajud R, et al. ADHD diagnosis and timing of medication initiation among children aged 3 to 5 years. *JAMA Netw Open*. 2025;8(8):e2529610. doi:10.1001/jamanetworkopen.2025.29610

eTable 1. List of codes used for well care visits and immunizations
eTable 2. List of codes used for ADHD diagnosis and ADHD medications
eTable 3. List of codes used for developmental/behavioral comorbid conditions
eTable 4. List of codes for behavioral problems used in sensitivity analysis
eTable 5. Patient demographics of children seen in primary care at ages 3-5 years across 8 institutions (n=712,478)
eTable 6. Time from diagnosis to prescription among preschool-age children with ADHD, stratified by patient age at time of initial ADHD-related diagnosis
eFigure 1. Follow up of children 3-5 years who were prescribed ADHD medications
eFigure 2. Time from initial ADHD diagnosis to medication prescription, stratified by the type of initial ADHD diagnosis (symptom-level first vs. disorder-level first)

This supplemental material has been provided by the authors to give readers additional information about their work.

© 2025 Bannett Y et al. *JAMA Network Open*.

eTable 1. List of codes used for well care visits and immunizations

Concept id*/ CPT code	Name	Domain	Vocabulary
4088016	Child examination	Procedure	SNOMED
1012596	Immunization administration (includes percutaneous, intradermal, subcutaneous, or intramuscular injections)	Procedure	CPT4
1012599	Immunization administration by intranasal or oral route	Procedure	CPT4
90620/1	Meningococcal recombinant, serogroup B	Drug	CPT4
90633	Hepatitis A vaccine, pediatric/adolescent	Drug	CPT4
90647/8	Hemophilus influenza B vaccine (Hib)	Drug	CPT4
90651	Human Papillomavirus vaccine (HPV)	Drug	CPT4
90670	Pneumococcal conjugate vaccine, 13 valent	Drug	CPT4
90672	Influenza virus vaccine, quadrivalent, live	Drug	CPT4
90680	Rotavirus vaccine, pentavalent (Rotateq)	Drug	CPT4
90681	Rotavirus vaccine, attenuated (Rotarix)	Drug	CPT4
90686	Influenza virus vaccine, quadrivalent	Drug	CPT4
90696	Diphtheria, tetanus toxoids, acellular pertussis vaccine and poliovirus vaccine, inactivated (DTaP-IPV)	Drug	CPT4
90698	Diphtheria, tetanus toxoids, acellular pertussis vaccine, hemophilus influenza Type B, and poliovirus vaccine, inactivated (DTaP-HIB-IPV)	Drug	CPT4
90700	diphtheria, tetanus toxoids, acellular pertussis vaccine (DtaP)	Drug	CPT4
90707	Measles, mumps, and rubella vaccine (MMR)	Drug	CPT4
90710	Measles, mumps, rubella, and varicella vaccine (MMRV)	Drug	CPT4
90713	Poliovirus vaccine, inactivated (IPV)	Drug	CPT4
90714	Tetanus and diphtheria toxoids (Td)	Drug	CPT4
90715	Tetanus, diphtheria toxoids and acellular pertussis vaccine (Tdap)	Drug	CPT4
90716	Varicella virus vaccine, live	Drug	CPT4
90723	Diphtheria, tetanus toxoids, acellular pertussis vaccine, Hepatitis B, and poliovirus vaccine, inactivated (DtaP-HepB-IPV)	Drug	CPT4
90732	Pneumococcal polysaccharide vaccine, 23-valent	Drug	CPT4
90734	Meningicoccal conjugate vaccine, serogoups A, C, Y and W-135 (quadtivalent)	Drug	CPT4
90744	Hepatitis B vaccine, pediatric/adolescent	Drug	CPT4

*We included these concept ids and all of their descendants
CPT= Current Procedural Terminology

© 2025 Bannett Y et al. *JAMA Network Open*.

eTable 2. List of codes used for ADHD diagnosis and ADHD medications

Condition type	ICD-10 code	Concept id*	Condition/Medication name
**ADHD diagnosis**			
ADHD (disorder-level)	F90.0	35207262	Attention-deficit hyperactivity disorder, predominantly inattentive type
ADHD (disorder-level)	F90.1	35207263	Attention-deficit hyperactivity disorder, predominantly hyperactive type
ADHD (disorder-level)	F90.2	45552506	Attention-deficit hyperactivity disorder, combined type
ADHD (disorder-level)	F90.8	35207264	Attention-deficit hyperactivity disorder, other type
ADHD (disorder-level)	F90.9	35207265	Attention-deficit hyperactivity disorder, unspecified type
ADHD (symptom-level)	F90.9	45533124	Hyperactivity; Hyperkinesis
ADHD (symptom-level)	R41.840	45582710	Inattention; Attention and concentration deficit
ADHD (symptom-level)	R41.844	45539344	Executive function deficit
ADHD (symptom-level)	R45.87	45568136	Impulsiveness
**ADHD medications**			
**Stimulants**			
		705944	Methylphenidate
		731533	Dexmethylphenidate
		714785	Amphetamine
		719311	Dextroamphetamine
		709567	Lisdexamfetamine
**Non-stimulants**			
		1344965	Guanfacine
		1398937	Clonidine
		742185	Atomoxetine

*We included these concept ids and all of their descendants

© 2025 Bannett Y et al. *JAMA Network Open*.

eTable 3. List of codes used for developmental/behavioral comorbid conditions

Condition type	ICD-10 code	Condition name
Autism	F84.0	Autism spectrum disorder
Autism	F84.5	Asperger's syndrome
Autism	F84.8	Other pervasive developmental disorders
Autism	F84.9	Pervasive developmental disorder, unspecified
Autism	F94.8	Other childhood disorders of social functioning
Autism	F94.9	Childhood disorder of social functioning, unspecified
Autism	F80.82	Social pragmatic communication disorder
Anxiety	F40	Phobic anxiety disorders
Anxiety	F41	Other anxiety disorders
Anxiety	F93.0	Separation anxiety disorder of childhood
Anxiety	F94.0	Selective mutism
Depression	F32	Depressive episode
Depression	F33	Recurrent depressive disorder
Depression	F34	Persistent mood [affective] disorder
Depression	F39	Unspecified mood [affective] disorder
Disruptive behavior disorder	F91	Conduct disorder, Oppositional defiant disorder
Disruptive behavior disorder	F63.81	Intermittent Explosive Disorder
Global Developmental Delay/Intellectual disability	F88	Global Developmental Delay/Other disorders of psychological development
Global Developmental Delay/Intellectual disability	F89	Unspecified disorder of psychological development
Global Developmental Delay/Intellectual disability	F70	Mild intellectual disabilities
Global Developmental Delay/Intellectual disability	F71	Moderate intellectual disabilities
Global Developmental Delay/Intellectual disability	F72	Severe intellectual disabilities
Global Developmental Delay/Intellectual disability	F73	Profound intellectual disabilities
Global Developmental Delay/Intellectual disability	F79	Unspecified intellectual disabilities
Learning problem/ disability	F81	Specific developmental disorders of scholastic skills
Learning problem/ disability	Z55	Problems related to education and literacy
Learning problem/ disability	R48.0	Dyslexia and alexia

Language delay/ disorder	F80.0	Phonological disorder
Language delay/ disorder	F80.1	Expressive language disorder
Language delay/ disorder	F80.2	Mixed receptive-expressive language disorder
Language delay/ disorder	F80.81	Childhood onset fluency disorder
Language delay/ disorder	F80.89	Other developmental disorders of speech and language
Language delay/ disorder	F80.9	Developmental disorder of speech and language, unspecified
Language delay/ disorder	R47.89	Other speech disturbances
Language delay/ disorder	R47.9	Unspecified speech disturbances
Sleep problems	G47	Sleep disorders
Sleep problems	F51	Sleep disorders not due to a substance or known physiological condition
Sleep problems	Z72.82	Problems related to sleep
Sleep problems	Z73.81	Behavioral insomnia of childhood

© 2025 Bannett Y et al. *JAMA Network Open*.

eTable 4. List of codes for behavioral problems used in sensitivity analysis

Condition type	ICD-10 code	Condition name
Behavioral problem	F91	Temper tantrums / oppositional behavior / disruptive behavior
Behavioral problem	R45.4	Irritability / anger
Behavioral problem	R45.5	Aggressive outburst / Hostility
Behavioral problem	R45.6	Violent behavior
Behavioral problem	R46.89	Behavior concern / behavior problem in child / aggression
Behavioral problem	Z72.810	Child and adolescent antisocial behavior, behavior problem at school

© 2025 Bannett Y et al. *JAMA Network Open*.

eTable 5. Patient demographics of children seen in primary care at ages 3-5 years across 8 institutions (n=712,478)

	Site A N = 310,150	Site B N = 95,619	Site C N = 159,989	Site D N = 56,714	Site E N = 29,837	Site F N = 44,152	Site G N = 5,041	Site H N = 10,976	Overall N=712,478
**Age at First Encounter**									
Median (IQR)	3.18 (3.03, 4.09)	3.25 (3.04, 4.23)	3.17 (3.04, 4.02)	3.47 (3.12, 4.28)	3.44 (3.11, 4.34)	3.27 (3.04, 4.38)	3.39 (3.08, 4.45)	3.29 (3.08, 4.07)	3.22 (3.04, 4.13)
**Sex (n, %)**									
Female	151,271 (48.8%)	46,234 (48.4%)	77,317 (48.3%)	27,488 (48.5%)	14,568 (48.8%)	21,444 (48.6%)	2,344 (46.5%)	5,213 (47.5%)	345,879 (48.5%)
Male	158,879 (51.2%)	49,385 (51.6%)	82,672 (51.7%)	29,226 (51.5%)	15,269 (51.2%)	22,708 (51.4%)	2,697 (53.5%)	5,763 (52.5%)	366,599 (51.5%)
**Race/ethnicity (n, %)**									
Hispanic	104,703 (33.8%)	18,342 (19.2%)	15,338 (9.6%)	8,678 (15.3%)	1,855 (6.2%)	4,871 (11.0%)	2,047 (40.6%)	4,999 (45.5%)	160,833 (22.6%)
Non-Hispanic Asian	20,446 (6.6%)	4,601 (4.8%)	8,627 (5.4%)	4,135 (7.3%)	658 (2.2%)	8,424 (19.1%)	318 (6.3%)	531 (4.8%)	47,740 (6.7%)
Non-Hispanic Black	46,732 (15.1%)	21,875 (22.9%)	40,986 (25.6%)	30,196 (53.2%)	15,940 (53.4%)	1,179 (2.7%)	1,651 (32.8%)	3,043 (27.7%)	161,602 (22.7%)
Non-Hispanic Multiple Races	8,395 (2.7%)	3,214 (3.4%)	6,177 (3.9%)	4,292 (7.6%)	1,416 (4.7%)	1,562 (3.5%)	139 (2.8%)	511 (4.7%)	25,706 (3.6%)
Non-Hispanic White	102,720 (33.1%)	40,308 (42.2%)	73,588 (46.0%)	8,701 (15.3%)	9,375 (31.4%)	10,109 (22.9%)	591 (11.7%)	1,284 (11.7%)	246,676 (34.6%)
Non-Hispanic Other	6,940 (2.2%)	4,343 (4.5%)	195 (0.1%)	91 (0.2%)	310 (1.0%)	3,909 (8.9%)	229 (4.5%)	475 (4.3%)	16,492 (2.3%)
Unknown	20,214 (6.5%)	2,936 (3.1%)	15,078 (9.4%)	621 (1.1%)	283 (0.9%)	14,098 (31.9%)	66 (1.3%)	133 (1.2%)	53,429 (7.5%)
**Insurance Plan (n, %)**									
Private	181,751 (58.6%)	53,362 (55.8%)	97,798 (61.1%)	8,257 (14.6%)	234 (0.8%)	36,862 (83.5%)	859 (17.0%)	1,345 (12.3%)	380,468 (53.4%)
	106,068 (34.2%)	41,486 (43.4%)	57,127 (35.7%)	47,081 (83.0%)	1,826 (6.1%)	7,290 (16.5%)	3,445 (68.3%)	9,222 (84.0%)	273,545 (38.4%)

© 2025 Bannett Y et al. *JAMA Network Open*.

Public	22,331 (7.2%)	771 (0.8%)	5,064 (3.2%)	1,376 (2.4%)	27,777 (93.1%)	0 (0.0%)	737 (14.6%)	409 (3.7%)	58,465 (8.2%)
Other/Unknown	181,751 (58.6%)	53,362 (55.8%)	97,798 (61.1%)	8,257 (14.6%)	234 (0.8%)	36,862 (83.5%)	859 (17.0%)	1,345 (12.3%)	380,468 (53.4%)
**Patients with at least 1 ADHD diagnosis at age 4-5 years (n, %)**	3,857 (1.2%)	1,886 (2.0%)	1,672 (1.0%)	934 (1.6%)	918 (3.1%)	202 (0.5%)	127 (2.5%)	112 (1.0%)	9,708 (1.4%)

© 2025 Bannett Y et al. *JAMA Network Open*.

eTable 6. Time from diagnosis to prescription among preschool-age children with ADHD, stratified by patient age at time of initial ADHD-related diagnosis

	Age at initial ADHD diagnosis			
	**3 Years** (N=376)	**4 Years** (N=2405)	**5 Years** (N=6927)	**Overall** (N=9708)
**Prescribed medication before age 7** (n, %)				
**Yes**				
Median (IQR) in days	390.5 (204.2, 672.0)	28.0 (0.0, 289.2)	0.0 (0.0, 55.0)	2.0 (0.0, 127.0)
≤30 days	6 (1.6%)	785 (32.6%)	3301 (47.7%)	4092 (42.2%)
30-183 days	55 (14.6%)	238 (9.9%)	866 (12.5%)	1159 (11.9%)
>183 days (6 months)	203 (54.0%)	527 (21.9%)	643 (9.3%)	1373 (14.1%)
**No**	112 (29.8%)	855 (35.6%)	2117 (30.6%)	3084 (31.8%)

© 2025 Bannett Y et al. *JAMA Network Open*.

eFigure 1. Follow up of children 3-5 years who were prescribed ADHD medications (n=6624)

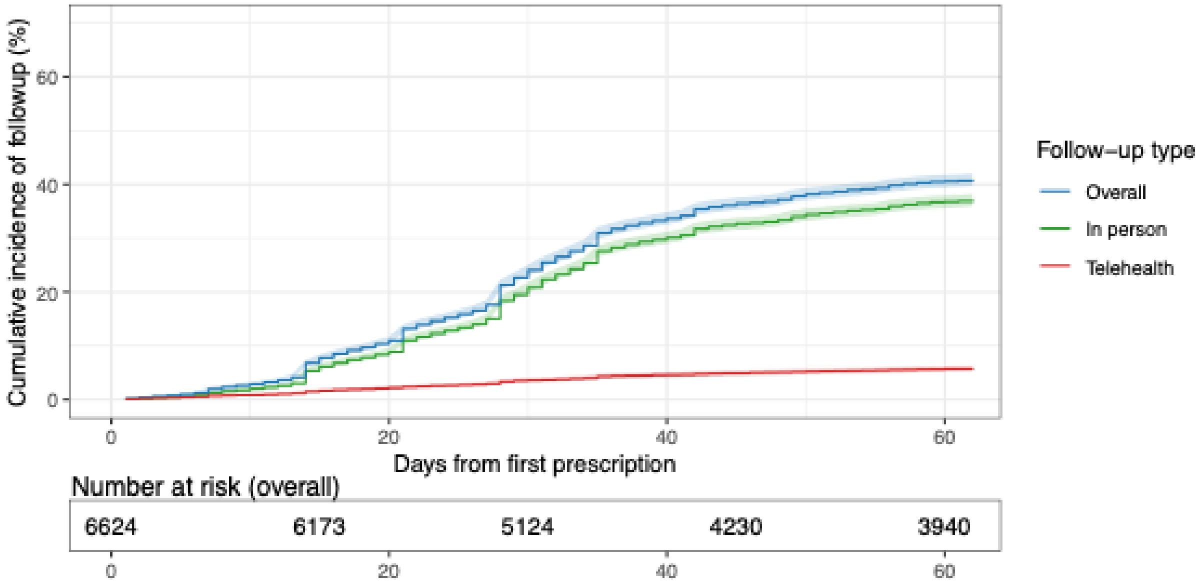

© 2025 Bannett Y et al. *JAMA Network Open*.

eFigure 2. Time from initial ADHD diagnosis to medication prescription, stratified by the type of initial ADHD diagnosis (symptom-level first vs. disorder-level first)

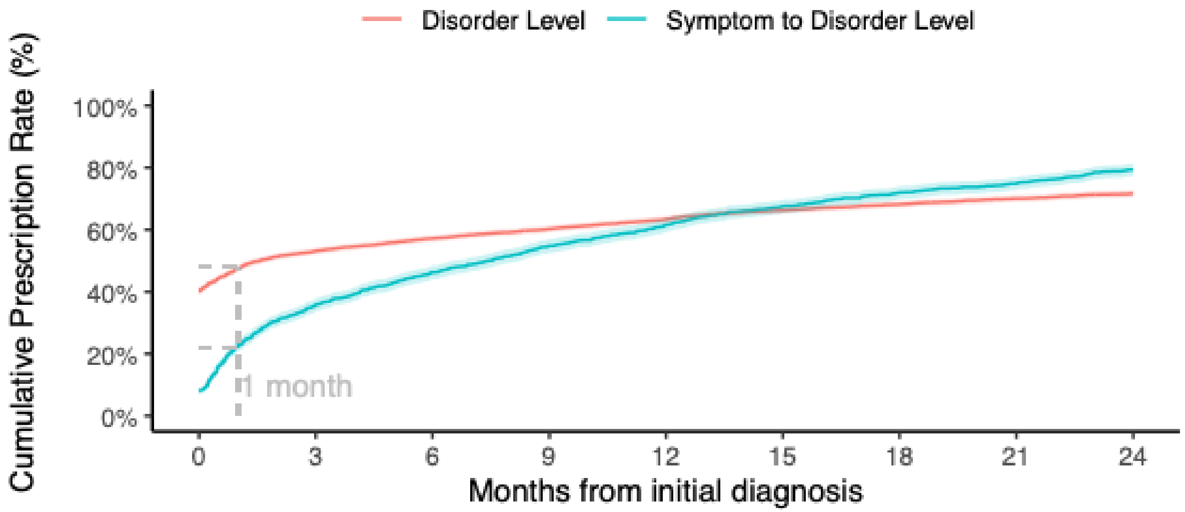

Disorder Level

At Risk	7728	3356	2941	2580	2265	1616	1129	761	405
Events	3109	4058	4345	4548	4747	4909	4987	5045	5078

Symptom to Disorder Level

At Risk	1978	1244	1028	838	693	530	412	307	213
Events	160	703	906	1067	1191	1293	1363	1401	1451

© 2025 Bannett Y et al. *JAMA Network Open*.
