# Supplementary material for: ADHD Diagnosis and Timing of Medication Initiation Among Children Aged 3 to 5 Years
Source: JAMA Netw Open. 2025 Aug 29;8(8):e2529610. doi: 10.1001/jamanetworkopen.2025.29610 (PMC12397892; doi:10.1001/jamanetworkopen.2025.29610)
Supplement: Supplement 2. — Data Sharing Statement [file jamanetwopen-e2529610-s002.pdf]

Data Sharing Statement

Bannett. ADHD Diagnosis and Timing of Medication Initiation Among Children Aged 3 to 5 Years. *JAMA Netw Open*. Published August 29, 2025.
doi:10.1001/jamanetworkopen.2025.29610

Data
Data available: No

Additional Information
Explanation for why data not available: The datasets generated and analyzed in the current study contain protected patient health information and are therefore not publicly available.
